# Protein kinase B (AKT) upregulation and Thy-1-α_v_β_3_ integrin-induced phosphorylation of Connexin43 by activated AKT in astrogliosis

**DOI:** 10.1186/s12974-022-02677-7

**Published:** 2023-01-06

**Authors:** Ramón Pérez-Núñez, Alejandro Chamorro, María Fernanda González, Pamela Contreras, Rocío Artigas, Alejandro H. Corvalán, Brigitte van Zundert, Christopher Reyes, Pablo R. Moya, Ana María Avalos, Pascal Schneider, Andrew F. G. Quest, Lisette Leyton

**Affiliations:** 1 Department of Cell and Molecular Biology, Cellular Communication Laboratory, Center for Studies On Exercise, Metabolism and Cancer (CEMC), Instituto de Ciencias Biomédicas (ICBM), Facultad de Medicina, Universidad de Chile, 838-0453 Santiago, Chile; 2 Advanced Center for Chronic Diseases (ACCDiS), Facultad de Medicina, Universidad de Chile, 838-0453 Santiago, Chile; 3 Advanced Center for Chronic Diseases (ACCDiS), Facultad de Medicina, Pontificia Universidad Católica de Chile (PUC), 833-1150 Santiago, Chile; 4 Department of Hematology and Oncology, Facultad de Medicina, Pontificia Universidad Católica de Chile (PUC), 833-1150 Santiago, Chile; 5 Institute of Biomedical Sciences (ICB), Faculty of Medicine & Faculty of Life Sciences, Universidad Andres Bello, 837-0186 Santiago, Chile; 6 Department of Neurology, University of Massachusetts Chan Medical School, Worcester, MA 01655 USA; 7 Instituto de Fisiología, Centro Interdisciplinario de Neurociencia de Valparaíso (CINV), Facultad de Ciencias, Universidad de Valparaíso, Valparaíso, Chile; 8 Facultad de Ciencias de la Salud, Instituto de Ciencias Biomédicas, Universidad Autónoma de Chile, Santiago, Chile; 9 Department of Biochemistry, University of Lausanne, 1066 Epalinges, Switzerland

**Keywords:** Brain damage, Inflammation, Astrogliosis, ALS model, Bioinformatics analysis, PI3K/AKT signaling pathway, Connexin43

## Abstract

**Background:**

In response to brain injury or inflammation, astrocytes undergo hypertrophy, proliferate, and migrate to the damaged zone. These changes, collectively known as "astrogliosis", initially protect the brain; however, astrogliosis can also cause neuronal dysfunction. Additionally, these astrocytes undergo intracellular changes involving alterations in the expression and localization of many proteins, including α_v_β_3_ integrin. Our previous reports indicate that Thy-1, a neuronal glycoprotein, binds to this integrin inducing Connexin43 (Cx43) hemichannel (HC) opening, ATP release, and astrocyte migration. Despite such insight, important links and molecular events leading to astrogliosis remain to be defined.

**Methods:**

Using bioinformatics approaches, we analyzed different Gene Expression Omnibus datasets to identify changes occurring in reactive astrocytes as compared to astrocytes from the normal mouse brain. In silico analysis was validated by both qRT-PCR and immunoblotting using reactive astrocyte cultures from the normal rat brain treated with TNF and from the brain of a hSOD1^G93A^ transgenic mouse model. We evaluated the phosphorylation of Cx43 serine residue 373 (S373) by AKT and ATP release as a functional assay for HC opening. In vivo experiments were also performed with an AKT inhibitor (AKTi).

**Results:**

The bioinformatics analysis revealed that genes of the PI3K/AKT signaling pathway were among the most significantly altered in reactive astrocytes. mRNA and protein levels of PI3K, AKT, as well as Cx43, were elevated in reactive astrocytes from normal rats and from hSOD1^G93A^ transgenic mice, as compared to controls. In vitro, reactive astrocytes stimulated with Thy-1 responded by activating AKT, which phosphorylated S373Cx43. Increased pS373Cx43 augmented the release of ATP to the extracellular medium and AKTi inhibited these Thy-1-induced responses. Furthermore, in an in vivo model of inflammation (brain damage), AKTi decreased the levels of astrocyte reactivity markers and S373Cx43 phosphorylation.

**Conclusions:**

Here, we identify changes in the PI3K/AKT molecular signaling network and show how they participate in astrogliosis by regulating the HC protein Cx43. Moreover, because HC opening and ATP release are important in astrocyte reactivity, the phosphorylation of Cx43 by AKT and the associated increase in ATP release identify a potential therapeutic window of opportunity to limit the adverse effects of astrogliosis.

**Supplementary Information:**

The online version contains supplementary material available at 10.1186/s12974-022-02677-7.

## Background

Astrocytes, the most abundant glial cell type in the central nervous system (CNS), become reactive upon brain damage: e.g., stroke, neurodegenerative diseases such as amyotrophic lateral sclerosis (ALS), or traumatic damage [66]. During the progression of reactivity, astrocytes proliferate, undergo morphological changes, migrate to the damaged zone and form a glial scar [20, 60]. Altogether, this process is known as astrogliosis or reactive gliosis and, although required to protect the brain, it represents a major impediment to nerve regeneration [21, 64, 65]. In addition, reactive astrocytes undergo changes in the expression and localization of cell surface proteins to sense extracellular matrix (ECM) molecules and proteins in other cell types [55]. Despite all the existing studies that have contributed to our current understanding of astrogliosis, gaps still remain, and a more thorough comprehension of this process is required.

Interestingly, the newly expressed proteins in reactive astrocytes include integrins [37], which form multiprotein complexes and generate points of adhesion to the ECM called focal adhesions (FA) [18, 24]. In this context, we have reported the direct interaction of α_v_β_3_ integrin in astrocytes with the neuronal glycoprotein Thy-1(CD90) [28, 30, 41]. Thy-1 has diverse functions [32, 40, 68] and its expression in neurons significantly increases in patients with inflammatory diseases [48, 71]. In the framework of the present study, a relevant function of the Thy-1-α_v_β_3_ integrin association is its involvement in neuron–astrocyte communication [30]. In neurons, Thy-1 signals via the Csk-binding protein (CBP) and the Src tyrosine kinase to induce axon retraction [29, 45]. Alternatively, the α_v_β_3_ integrin in astrocytes signals through various kinases and protein phosphorylation events to stick and peel-off FA from the ECM, thus allowing cells to move [reviewed in [40]].

Interestingly, the Thy-1-α_v_β_3_ integrin interaction induces changes in astrocytes in vitro*,* which are comparable to those reported in vivo when astrogliosis is triggered [5, 14, 41]. These changes include increased cell surface protein levels, cell adhesion and migration. Molecular mechanisms governing Thy-1-induced astrocyte migration include hemichannel (HC) opening, ATP release, activation of purinergic P2X7 receptors (P2X7Rs), and Ca^2+^ influx into the cytosol [2, 6, 27, 37]. The HCs involved in this signaling pathway are those formed by Connexin43 (Cx43) and Pannexin1 [37], two proteins whose expression is upregulated when astrocytes are exposed to inflammatory signals [34, 37, 54].

Under inflammatory conditions, such as those induced by conditioned media from activated microglia, astrocytes increase their permeability by opening Cx43 HCs, and decrease communication through Cx43 gap junctions [39, 54]. Moreover, cytokines or neuroinflammation also increase Cx43 protein levels and HC activity in astrocytes in vivo [1], and in astrocytes treated with the amyloid-β-peptide, they help to maintain the astrocyte reactive phenotype by releasing molecules, such as ATP and glutamate [10, 12, 50]. Therefore, HC, rather than gap junctions, participate in the reactive gliosis process.

To identify up- and downregulated molecules in reactive versus non-reactive astrocytes, we used a bioinformatics approach and analyzed various Gene Expression Omnibus (GEO) datasets. The chosen GEO sets covered a wide range of insults, because reportedly, every type of injury generates different responses in at least 50% of the genes that are upregulated following damage [73]. Here, we show that some of the most altered genes in reactive astrocytes were those related to the PI3K/AKT, Regulation of the Actin Cytoskeleton, and FA signaling pathways. Our previous studies reported the activation of PI3K and AKT in astrocytes downstream of the Thy-1-α_v_β_3_ integrin interaction [36]; however, changes in protein expression and particularly, upregulation of these genes in an inflammatory context in astrocytes has never been observed previously. Thus, we set out to validate the bioinformatics findings and to search for molecular targets downstream of the PI3K/AKT pathway. One possibility was Cx43, which is known to be phosphorylated on serine residue 373 (S373) by AKT [51]. Moreover, in osteocytes stimulated by fluid flow shear stress, this AKT-mediated phosphorylation event leads to the activation and opening of Cx43 HCs, thereby implicating this sequence in transducing mechanical signals in bone cell responses [9].

Here, we used neonatal rat astrocytes, treated or not with TNF, as well as astrocytes obtained from a well-studied ALS mouse model (hSOD1^G93A^, gene encoding superoxide dismutase 1), as sources of reactive astrocytes [37]. Under inflammatory conditions, mRNA and protein levels of both PI3K and AKT were upregulated. In addition, the Thy-1-α_v_β_3_ integrin interaction in reactive astrocytes led to the phosphorylation of Cx43 on S373 by AKT. Moreover, using in vitro and in vivo models, we show that S373Cx43 was phosphorylated under proinflammatory conditions and such phosphorylation was inhibited by AKTi. Additionally, in vitro S373Cx43 phosphorylation was required for ATP release to the extracellular medium. Importantly, these signaling cascades are key players in the molecular sequence of events leading to cell migration [2, 13, 37], which is an important feature of reactive astrocytes.

## Methods

### Animals

Care and use of rodents were in accordance with the protocols approved by the bioethical Committees of Universidad de Chile, Universidad Andrés Bello, and Universidad de Valparaíso. Wistar neonatal rats (P0-P1) were obtained from the animal facility at the Faculty of Medicine, Universidad de Chile. Hemizygous transgenic B6SJL mice for mutant human SOD1 (hSOD1^G93A^) and wild-type human SOD1 (hSOD1^WT^, used as control) were originally obtained from Jackson Laboratories (Bar Harbor, USA). Transgenes were identified by the polymerase chain reaction, as previously reported [22, 58, 61, 70]. For the in vivo assays, wild-type C57BL/6J male mice were obtained from the animal facility at the Faculty of Sciences, Universidad de Valparaíso.

### Cell culture

Primary astrocytes from 0- to 1-day-old wild-type rats or hSOD1^G93A^ and hSOD1^WT^ transgenic mice were obtained and maintained as previously described [37]. The mouse catecholaminergic neuronal cell line CAD [52] was maintained in DMEM/F12 medium (Gibco, Life Technologies, Grand Island, NY), supplemented with 10% FBS (Biological Industries, Cromwell, CT, USA) and 1% penicillin/streptomycin at 37 °C and 5% CO_2_. Alternatively, CAD cells were differentiated in serum-free DMEM/F12 medium (SFM) containing sodium selenite (50 ng/ml). To promote a proinflammatory environment, rat primary astrocytes were stimulated with TNF (10 ng/ml) for 48 h [2]. The source of TNF was a recombinant Fc-fusion protein (Fc-mTNF) affinity purified on Protein A-Sepharose beads, as for the other Fc-fusion proteins [13].

### Thy-1-Fc and TRAIL-R2-Fc preparation

Thy-1-Fc and TRAIL-R2-Fc (used as a negative control) fusion proteins were obtained as previously described [2, 36]. Before stimulation, Thy-1-Fc and TRAIL-R2-Fc were incubated with Protein A in a 10:1 ratio, while rotating gently on a shaker for 1 h, at 4 °C. Prior to each experiment involving stimulation with Thy-1-Fc:Protein A or TRAIL-R2-Fc:Protein A in SFM, astrocytes were serum-deprived for 30 min in DMEM/F12 medium. We have used TRAIL-R2-Fc in our studies as a control for Thy1-Fc for many years. We continued to use it on the rationale that the phenotype of cells stimulated with TRAILR2-Fc is like that of untreated cells; for example, when measuring cell polarization, adhesion, or migration [6, 36].

### Bioinformatics analysis

#### Transcriptome array data set selection and data preprocessing

The search and selection of four series of datasets (GSE35338, GSE40857, GSE73022, GSE28731) was made using the Gene Expression Omnibus (GEO) repository [8, 23, 53, 73], and the keywords “reactive gliosis”. The in silico analysis was performed using these datasets, which were obtained by various treatments, such as: Optic nerve head crush (ONC), middle cerebral artery occlusion with or without lipopolysaccharide treatment, and TNF (Additional files: Table S1). We used these microarrays to identify differentially expressed genes and to investigate the molecular mechanisms of reactive gliosis. Since the GSE40857 dataset was the largest and the one showing the greater number of altered genes, we chose it for the detailed analyses. The dataset was obtained comparing astrocytes from the normal optic nerve head to those undergoing astrocytosis in response to ONC. The transcriptome arrays were run on the GeneChip Mouse Genome 430A Affymetrix 2.0 Array (GPL8321), a single array that includes 20,690 probes, which represent approximately 14,000 well-characterized mouse genes (http://www.affymetrix.com). We downloaded 55 transcriptomic arrays as. CEL files and preprocessed the data. First, we assessed the quality of the assays, and normalized the data using the quantile’s method. All the analyses were performed using the 3.6.1 version of the R Software (https://cran.rproject.org/). The exploratory step of our analysis was based on the principal component analysis (PCA), to visualize sample distribution and clustering of the Optical Nerve Crush (ONC) condition versus the Contralateral Control (CC) condition. This algorithm reduces the dimensionality of the data, while maintaining the directions with the highest variability. Sample distribution was plotted according to these directions, or principal components (PC). The samples were expected to group according to their main differences or similarities [56].

#### Differential expression analysis (DEA)

GSE40857 PCA showed that the samples were distributed in two main groups, except for 5 replicates of the 3 months-CC treatment, and 3 replicates of the 3 months-ONC treatment. Since these 8 samples formed a different group, and were separated from the rest by PCA, we decided to treat them as outliers and therefore, removed them from the analysis because they were not representative of the corresponding condition. Therefore, we ran a DEA using the Linear Models for Microarray Data contained in the R limma package [57], based on the expression profiling of the remaining 47 samples. P-values were corrected using the Benjamini–Hochberg method [11]. Finally, 5991 differentially expressed genes (DEGs) were obtained (*p* < 0.05) and separately subjected to Functional Enrichment Analysis and Gene Networks construction, using the BinGO 3.0.3 app and the Wikipathways 3.3.7 plugin, respectively, both of which are available in the 3.7.2 version of the Cytoscape software [63].

### Quantitative real-time reverse transcription PCR (qRT-PCR)

Primary rat astrocytes treated or not with TNF were lysed directly on the plates after 48 h, adding 1 ml of TRIzol™ Reagent (Cat. 15596-026, ThermoFisher Scientific, Waltham, MA, USA) per 10 cm^2^ of area to ensure sufficient cell disruption, according to the manufacturer's instructions. Then, concentration and purity of the isolated RNA were determined by spectrophotometry at 260 and 280 nm. RNA quality was verified by 1% agarose gel electrophoresis. Reverse transcription was performed with 1 µg of RNA using a kit, according to the manufacturer’s indications (SuperScript® III First-Strand Synthesis System, Cat. 18,080–051, ThermoFisher Scientific). The qPCR reaction was carried out with the complementary DNA (cDNA), using the SYBR™ Green PowerUp™ Master Mix kit (Cat. A25776, ThermoFisher Scientific) as a fluorescent agent, together with the following specific primers for each gene: *PI3K* FW 5′-AGA GGG GTA CCA GTA CAG AGC-3′, RV 5′-CCC CCA AGT GTA GGT CGA TG-3′; *AKT* FW 5′-TCT ATG GCG CTG AGA TTG TG-3′, RV 5′-CTT AAT GTG CCC GTC CTT GT-3′; *Cx43* FW 5′-GAA CAC GGC AAG GTG AAG AT-3′, RV 5′-GAG CGA GAG ACA CCA AGG AC-3′; and *GAPDH* FW 5′-AAC TCC CAC TCT TCC ACC TT-3′, RV 5′-TTA CTC CTT GGA GGC CAT GT-3′ (Integrated DNA Technologies, Coralville, IA, USA). PCR was performed on a real-time thermocycler (Mx3000P, Stratagene, San Diego, CA, USA) and the results were analyzed using the MxPro v2.0 program. The expression was quantified using the 2^−∆∆Ct^ method [44] and values were normalized to the quantity of GAPDH as a reference housekeeping gene.

### Western blot

Protein extracts were obtained from astrocyte cultures using the radioimmunoprecipitation assay (RIPA) buffer (150 mM NaCl, 0.1% sodium dodecyl sulphate, 0.5% sodium deoxycholate, 0.1% Triton X-100, in 50 mM Tris–HCl pH 8.0), supplemented with a protease and phosphatase inhibitor cocktail (Biotool, Houston, TX, USA). Extracts were separated by 10% SDS-PAGE and transferred to nitrocellulose membranes (Millipore, Billerica, USA). The membranes were blocked with 3% Bovine Serum Albumin, 0.1% Tween-20 in TBS and subsequently incubated with the following primary antibodies: anti-Cx43 (1:2000, Cat. 13–8300, ThermoFisher Scientific, Waltham, MA, USA), anti-phosphoS373Cx43 (1:1000, Cat. PA5-64670, ThermoFisher Scientific), mouse anti-PI3K (1:2000, Cat. MA1-74783, ThermoFisher Scientific), rabbit anti-AKT (1:2000, Cat. 9272S, Cell Signaling Technology, Danvers, MA, USA), rabbit anti-phosphoS473AKT (1:1000, Cat. 4060S, Cell Signaling Technology), and mouse anti-Hsp90 α/β (1:2000, Cat. Sc-13119, Santa Cruz Biotechnologies, Dallas, TX, USA). Membranes were then washed and incubated with horseradish peroxidase-conjugated goat anti-rabbit IgG (1:5000; Abbexa, Cambridge, UK) or goat anti-mouse IgG (1:5000; Abbexa) for 2 h at room temperature. Bands were visualized with a chemiluminescence kit (Pierce, Thermo Scientific, Rockford, IL, USA), according to the manufacturer’s instructions, and detected using the Discovery 12iC model chemiluminescence system from Ultralum (Claremont, CA, USA). Densitometric analysis of the immunoreactive bands was performed with the Gel Pro Analyzer 4.0 software (Media Cybernetics, Inc.) and the values were normalized to the corresponding loading control.

### Flow cytometry

For the viability assays, CAD cells were recovered from culture dishes with a solution of 25 mM ethylenediaminetetraacetic acid (EDTA) in PBS. The cells were washed twice with PBS and resuspended in 0.5 ml of PBS (1–10 × 10^6^/ml). Then, Fixable Viability Dye eFluor™ 780 (FVD, Cat. 65–0865, ThermoFisher Scientific) was added (1 µl/ml), incubating for 30 min at 2–8 °C. Cells were then washed twice with flow cytometry staining buffer and fixed in 2% paraformaldehyde in PBS. Data were acquired on a FACSort (Becton Dickinson, Franklin Lakes, NJ, USA) flow cytometer and analyzed using the version 2.9 of the WinMD program. Analyses were performed on at least 10,000 events from the total population, and all the data were corrected by the control basal levels.

### Extracellular ATP measurements

Primary astrocytes (5 × 10^4^) were seeded per well in 48-well plates. After 24 h, cells were incubated in SFM containing 100 μM of the exonuclease inhibitor ARL-67156 (Santa Cruz Biotechnologies, Dallas, TX, USA) for 30 min at 37 °C. Then, the cells were stimulated with 2 µg of Thy-1-Fc per well, for 10 min. Where indicated, cells were incubated for 30 min with an AKT-inhibitor (3 µM AKTi, AKT inhibitor VIII, Merck Millipore, Burlington, MA, USA) or a PI3K-inhibitor (3 µM LY294002, Sigma-Aldrich Co., St. Louis, MO, USA). Next, the culture medium was recovered and centrifuged for 5 min at 1000 × *g*. The supernatants were incubated in the dark with 50 μl CellTiter-Glo® reaction mix (Promega, Madison, WI, USA). Luminescence intensity was determined in a Synergy2 multi-mode reader (Biotek Instruments, Inc., Winooski, VT, USA), and the values were calculated using a calibration curve obtained with ATP concentrations of 0.01, 0.1, 1 and 10 μM.

### Indirect immunofluorescence

Primary rat astrocytes and astrocytes derived from hSOD1 transgenic mice were seeded on 12-mm coverslips and left to adhere for 24 h. Rat astrocytes were stimulated with TNF for 48 h or left untreated. The cells were then incubated for 30 min with 3 µM AKTi and stimulated with Thy-1-Fc for 10 min. Afterwards, they were washed, fixed, and stained with rabbit anti-phosphoCx43 (1:200, ThermoFisher Scientific) antibody, followed by a secondary antibody conjugated to IF488 (1:400, Abbexa) and 4′,6-diamidino-2-phenylindole (DAPI) (1:5000, ThermoFisher Scientific) for nuclear staining. Phalloidin conjugated to rhodamine was used to visualize F-actin. Samples were analyzed using a C2 + confocal microscope (Nikon, Tokyo, Japan), with a 40 × /1.40 objective and the NIS-Elements software. Quantification was performed using the Fiji ImageJ software, where each image was analyzed in 8 bits. An intensity scale from 0 to 255 was used to measure the mean fluorescence intensity of pCx43. At least 20 cells were analyzed per condition, per experiment.

### In vivo assays

Surgical procedures were performed in accordance with the Guide for the Care and Use of Laboratory Animals (National Institutes of Health) and approved by the Institutional Bioethics Committee at Universidad de Valparaíso. C57BL/6J male mice weighing 20–25 g were anesthetized with a ketamine/xylazine cocktail (100 mg/kg/10 mg/kg) and placed in a Kopf stereotaxic apparatus (Kopf, CA, USA), with controlled body temperature (37 °C), using a heating pad (CMA, Sweden). Mice were bilaterally injected in the cortex using a Microinjector Unit Model 5000 (Kopf) with a Hamilton Syringe (33 gauge). Coordinates of injection site were (in mm) AP -1 ± 0.5 relative to Bregma, and DV-1.3 relative to the skull surface. AKTi (60 nmol, 10% DMSO) or vehicle (10% DMSO) dissolved in 0.5 μl of artificial cerebrospinal fluid (in mM: 125 NaCl, 2.5 KCl, 2 CaCl_2_, 1 MgCl_2_, 25 NaHCO_3_, 1.25 NaH_2_PO_4_, and 25 glucose, conditioned to 95% O_2_, 5% CO_2_, pH 7.4) were infused into the prefrontal cortex at a rate of 0.1 μl/min. After infusion, the needle was left in place for 5 additional min and then slowly retracted. After monitored recovery, mice were returned to their home cage for 1 day. Mice were then intracardially perfused with 4% paraformaldehyde; brains were removed from the skull, post-fixed in 4% paraformaldehyde for 24 h and incubated in 30% sucrose (Merck, Millipore, Burlington, MA) for 48 h. Brain slices of 20 µm were obtained using a Leica cryostat (Leica Biosystem, Buffalo Grove, IL, USA) and kept in PBS until further processing. Subsequently, the different sections were transferred to 24-well plates. Then, the slices were washed twice with PBS, blocked and permeabilized for 1 h with 2% Bovine Serum Albumin and 0.5% Triton X-100 in PBS, and incubated with anti-phosphoCx43 (1:200, ThermoFisher Scientific) or mouse anti-Glial Fibrillary Acidic Protein (GFAP) (1:200, Sigma-Aldrich Co.) primary antibodies, during 24 h, followed by a 2 h incubation with secondary antibodies conjugated to IF488 or IF594 (1:400, Abbexa), and DAPI (1:5000, ThermoFisher Scientific). The different slices were mounted on 25-mm coverslips and analyzed using a C2 + confocal microscope (Nikon, Tokyo, Japan), with a 40 × /1.40 objective and the NIS-Elements software. Quantification using the Fiji ImageJ software was done as indicated for indirect immunofluorescence. An intensity scale from 0 to 255 was used to measure the mean fluorescence intensity of GFAP and pCx43. At least 20 cells were analyzed per experiment.

### In vitro test for neuronal damage

Primary astrocytes were cultured in DMEM/F12 under the following conditions: no TNF, TNF, and TNF + 3 μM AKTi for 48 h. The cells were then washed and fresh SFM was added. Astrocyte-conditioned medium (ACM) obtained 5 days post-stimulation was collected and filtered through 0.2-μm filters. The ACM was diluted by 50% in DMEM/F12, added to the differentiated CAD cells and incubated for 24 h. CAD cells cultured with serum-containing medium (SCM) and CAD cells cultured in differentiation medium (DMEM/F12 containing sodium selenite) were included as positive and negative controls, respectively. At the end of the experiment, a total of 6 images were obtained with a microscope (Oxion Inverso Biological Microscopes, Euromex microscopes, Holland, NL) using duplicates of each condition. Images were analyzed using the NeuronJ plugin of the ImageJ software, as previously reported by our group [29, 45].

### Statistical analyses

We used the GraphPad Prism 6 software (San Diego, CA, USA). Data indicate the mean ± standard error of the mean (s.e.m.) of results from at least three or more independent experiments, unless otherwise specified. Results were analyzed with the Mann–Whitney non-parametric test to compare the distributions of two groups, or by ANOVA with a Bonferroni post hoc test to compare multiple groups. The specific test used for the statistical analyses is indicated in each figure legend. *p*-values < 0.05 were considered statistically significant.

## Results

### Altered gene expression in reactive astrocytes

**Fig. 1:**
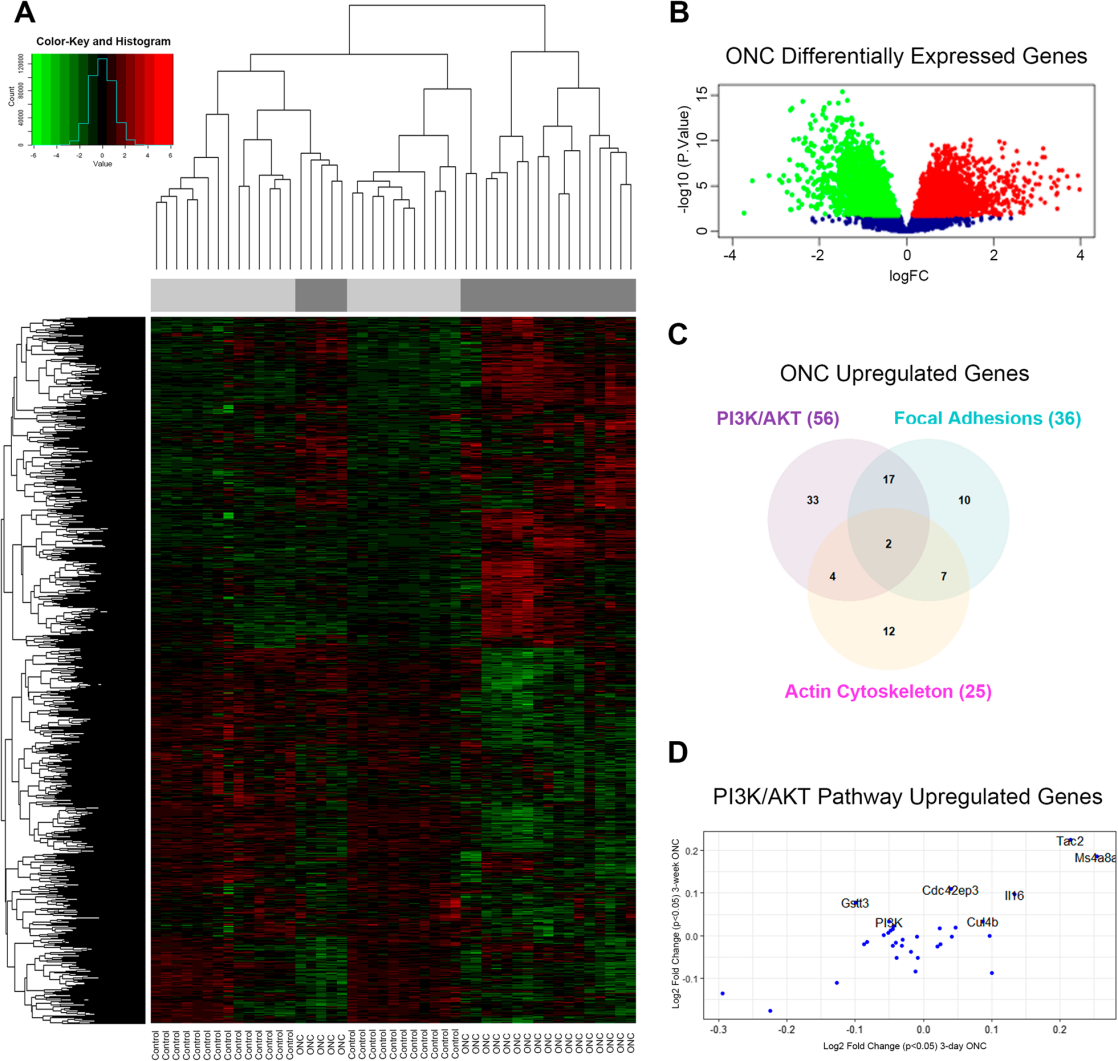
Hierarchical clustering and significance analysis of microarrays. **A** Heatmap of a representative GSE40857 dataset shows a dendrogram of genes to the left of the figure, which includes the 8617 most differentially expressed genes, before false discovery rate (FDR) correction. The dendrogram of samples at the top shows almost all the control samples grouping in one cluster (light grey bar), while only ONC samples appeared in the second cluster (dark grey bar), according to the PCA analysis, where the red and green colors represent high-scale and low-scale intensities, respectively (color-key bar). **B** Volcano plot of ONC-differentially expressed genes, which represents the number of up- and downregulated genes and the non-significantly changed gene proportions in red, green, and blue, respectively. **C** A Venn Diagram reveals differential gene activation and overlapping of upregulated genes in three main signaling pathways: PI3K/AKT, Regulation of Actin Cytoskeleton, and Focal Adhesions. The numbers in parentheses indicate the total number of upregulated genes in each one of these pathways. **D** Dot plot graph of PI3K/AKT pathway upregulated genes. Plot compares 3-day ONC versus 3-week ONC treatments

We first set out to study alterations in gene expression between reactive astrocytes and naïve astrocytes to identify new signaling molecules/pathways involved in astrogliosis. The bioinformatics analysis initially included four series of datasets (GSE35338, GSE40857, GSE73022, GSE28731) obtained after different cerebral insults or treatments: optic nerve head crush (ONC), middle cerebral artery occlusion with or without lipopolysaccharide treatment, and TNF (Additional file 1: Table S1). Detailed analysis of the datasets revealed a greater number of genes, both up- and downregulated, in three main signal transduction cascades: PI3K/AKT, Regulation of Actin Cytoskeleton and FA signaling (Additional file 2: Table S2). Our previous reports had confirmed that the “Regulation of Actin Cytoskeleton” and “FA” cascades were altered in reactive astrocytes [37, 38]. Thus, we focused here on validating the involvement of the PI3K/AKT pathway in the Thy-1-induced reactive astrocyte responses. As mentioned in the “Methods” section, we chose the GSE40857 dataset for a more detailed analysis. Hierarchical clustering results presented in a heatmap revealed the resulting sample and gene clusters (x and y axes, respectively), based on the probes that were differentially expressed between the ONC and control groups (Fig. 1A). Almost all the samples belonging to the same group were spontaneously clustered together (based on the expression of their most differentially expressed genes), indicating that the data from reactive astrocytes segregated from controls, irrespective of the treatment (Fig. 1A). We then created a Volcano plot to visually recognize genes with large fold changes that were statistically significant. This plot identified a similar number of up- and downregulated genes that were significantly different between quiescent and reactive astrocytes (Fig. 1B). The number of genes that were unique and shared among the three most affected pathways is summarized in a Venn diagram (Fig. 1C). A greater number of genes (56 in total) were identified in the PI3K/AKT interacting network, of which 33 were unique to this pathway. Interestingly, PI3K (NM011083, accession number in Table 1) was one of the 7 most highly upregulated genes, when comparing the 3-day ONC versus the 3-week ONC treatments in a dot plot (Fig. 1D). Therefore, our in silico and in vitro studies provide evidence that reactive astrocytes have > 8000 up- and downregulated genes, and the Cytoscape analysis grouped these genes mainly in three molecular interacting networks: PI3K/AKT, Regulation of Actin Cytoskeleton, and FA.

**Table 1:** Upregulated genes of the most altered pathways in reactive astrocytes

Accession number	Gene	Fold	*P* value
*PI3K/AKT signaling pathway*			
X75483	Cyclin A2	3.57	2.0E−07
AK013312	Cyclin B2	2.86	1.3E−03
NM007659	Cyclin-dependent kinase 1	2.82	2.9E−06
NM007629	Cyclin B1/predicted gene 5593	2.00	7.2E−04
AU015121	Cyclin B1	1.96	6.2E−03
NM009873	Cyclin-dependent kinase 6	1.67	1.5E−05
NM011146	Peroxisome proliferator activated receptor gamma	1.42	4.9E−05
BM935811	Integrin alpha 6	1.32	6.5E−08
NM010576	Integrin alpha 4	1.32	6.0E−03
AF091432	Cyclin E2	1.30	2.0E−04
NM016756	Cyclin-dependent kinase 2	1.18	5.4E−04
AF032460	BCL2-like 11 (apoptosis facilitator)	1.11	1.3E−03
NM007634	Cyclin F	1.10	1.3E−02
BB768208	Serum/glucocorticoid regulated kinase 3	1.03	6.7E−05
NM011905	Toll-like receptor 2	0.93	9.8E−03
BM120341	Integrin beta 1 (fibronectin receptor beta)	0.92	5.7E−06
NM007633	Cyclin E1	0.88	1.4E−02
NM008396	Integrin alpha 2	0.84	3.4E−03
NM016746	Cyclin C	0.73	5.7E−06
BC003828	RAS-related C3 botulinum substrate 1	0.70	1.0E−06
NM023243	Cyclin H	0.67	8.5E−09
U95826	Cyclin G2	0.62	1.7E−04
BB051001	Cyclin J	0.61	8.4E−04
BG070845	Cyclin-dependent kinase 12	0.60	1.1E−02
NM009870	Cyclin-dependent kinase 4	0.58	2.1E−05
BB623587	Integrin alpha 8	0.57	2.2E−02
NM011083	Phosphatidylinositol 3-kinase	0.54	1.1E−05
AF185285	Toll-like receptor 4	0.50	1.0E−02
NM133721	Integrin alpha 9	0.41	1.7E−02
NM010761	Cyclin D-type binding-protein 1	0.38	5.4E−03
AF124142	Thymoma viral proto-oncogene 3	0.35	1.4E−02
BG065754	Cyclin G1	0.32	1.8E−03
*Regulation of Actin Cytoskeleton*			
NM011146	Peroxisome proliferator activated receptor gamma	1.42	4.9E−05
BM238906	NCK associated protein 1 like	0.86	2.3E−02
BC027242	Vav 3 oncogene	0.74	7.7E−03
BE372352	ARP3 actin-related protein 3	0.55	8.4E−07
NM011083	Phosphatidylinositol 3-kinase	0.54	1.1E−05
NM011072	Profilin 1	0.54	2.9E−02
AW537308	p21 protein (Cdc42/Rac)-activated kinase 2	0.36	1.4E−02
AK014859	ARP2 actin-related protein 2	0.33	2.2E−05
*Focal Adhesions*			
BM935811	Integrin alpha 6	1.32	6.5E−08
NM010576	Integrin alpha 4	1.32	6.0E−03
NM009008	RAS-related C3 botulinum substrate 2	1.21	8.8E−04
BM246972	RAS related protein 1b	1.19	7.5E−09
BM940281	Mitogen-activated protein kinase 8	0.96	4.1E−04
BM120341	Integrin beta 1 (fibronectin receptor beta)	0.92	5.7E−06
NM008396	Integrin alpha 2	0.84	3.4E−03
BC027242	Vav 3 oncogene	0.74	7.7E−03
BC003828	RAS-related C3 botulinum substrate 1	0.70	1.0E−06
BB623587	Integrin alpha 8	0.57	2.2E−02
NM011083	Phosphatidylinositol 3-kinase	0.54	1.1E−05
NM011949	Mitogen-activated protein kinase 1	0.51	3.2E−05
BC011105	RAS-related protein-1a	0.47	8.0E−04
NM133721	Integrin alpha 9	0.41	1.7E−02
AW537308	p21 protein (Cdc42/Rac)-activated kinase 2	0.36	1.4E−02
AF124142	Thymoma viral proto-oncogene 3	0.35	1.4E−02
BC015289	Vasodilator-stimulated phosphoprotein	0.34	2.0E−02

The table contains all the differentially expressed genes (DEGs) that resulted from the GSE40857 analysis. Log Fold Change ratio (logFC) and adjusted *p*-values (corrected by the Benjamini–Hochberg method [35]) are shown for each gene. logFC > 0 and an adjusted *p*-value < 0.05 indicate that the gene is upregulated in the ONC group

### Validation of the PI3K/AKT pathway changes in reactive astrocytes

**Fig. 2:**
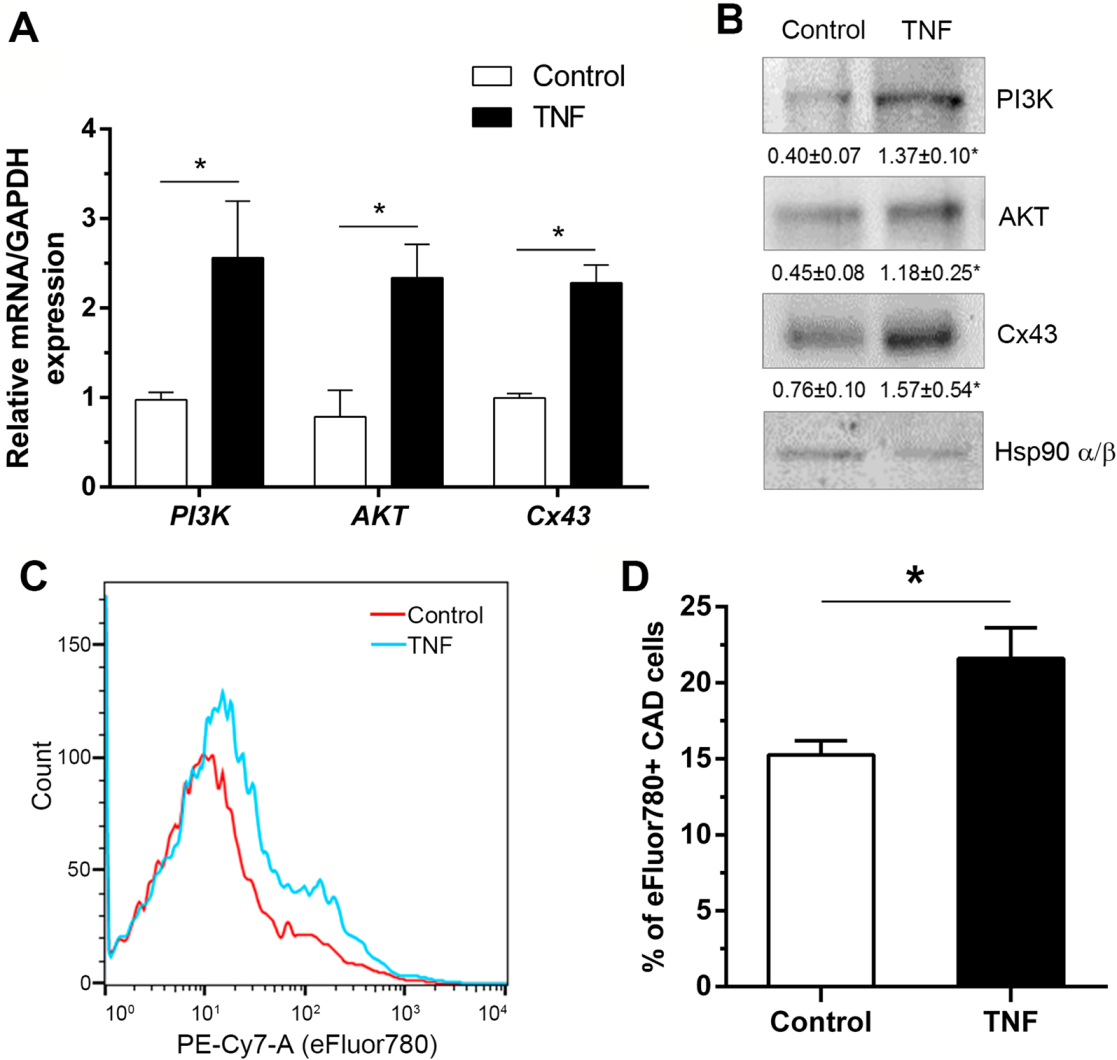
PI3K, AKT and Cx43 expression levels in reactive astrocytes. **A** Astrocytes were treated for 48 h with TNF (10 ng/ml) or left untreated (Control) and messenger RNA (mRNA) was extracted from the samples to measure PI3K, AKT and Cx43 mRNA levels by qRT-PCR. Values indicate fold increase normalized to control values. **B** Representative Western blots of PI3K, AKT, Cx43 and Hsp90 α/β (loading control) levels in non-treated astrocytes (Control) or astrocytes treated with TNF (TNF). Values indicate the ratio between the densitometric values of the bands and the respective Hsp90 α/β control values. **C** Histograms of neuronal cell viability (CAD cells) measured by flow cytometry following treatment for 72 h with the conditioned medium obtained from non-treated (Control, red line) or TNF-treated astrocytes (TNF, light blue line). **D** Quantification of the percentage of neuronal cell viability using eFluor780 in CAD cells, as described in **C**. Values are mean ± s.e.m. from 3 separate experiments, *p < 0.05 (Mann–Whitney test)

To validate the results of the bioinformatics analysis, we measured PI3K and AKT mRNA levels using mRNA extracts obtained from rat astrocytes, treated or not with TNF for 48 h. As a positive control, we used the Gap Junction protein alpha 1 (*GJA1*) gene encoding for Cx43, which is upregulated in TNF-treated astrocytes [37]. Results show that both PI3K and AKT mRNA levels increased in reactive astrocytes, compared to non-reactive controls (Fig. 2A). Likewise, Cx43 expression levels were also upregulated in TNF-treated astrocytes (Fig. 2A). We then measured protein levels in both reactive and non-reactive astrocytes and found significantly higher PI3K and AKT levels in TNF-treated reactive astrocytes, compared to the untreated controls (Fig. 2B). As expected, Cx43 mRNA and protein levels were also elevated in reactive astrocytes (Fig. 2B).

High Cx43 protein levels are indicative of astrocyte reactivity [37, 54]; however, in an in vitro astrogliosis model, additional functional proof of the reactivity process is required. In our previous report, we confirmed astrocyte reactivity by evaluating several molecular markers [37]. Here, we validated reactivity by testing whether conditioned medium obtained from TNF-treated astrocytes could harm and kill neurons, as has been widely reported [25, 43]. To this end, we measured viability of a neuronal-CAD cell line incubated for 72 h with the conditioned medium of reactive astrocytes. The percentage of eFluor780-positive cells estimated by flow cytometry was elevated, indicative of neuronal cell death after incubating CAD cells with the reactive astrocyte supernatant, as compared to the control condition (Fig. 2C, D).

### Phosphorylation of Cx43 by Thy-1 occurs through activation of the PI3K/AKT pathway

**Fig. 3:**
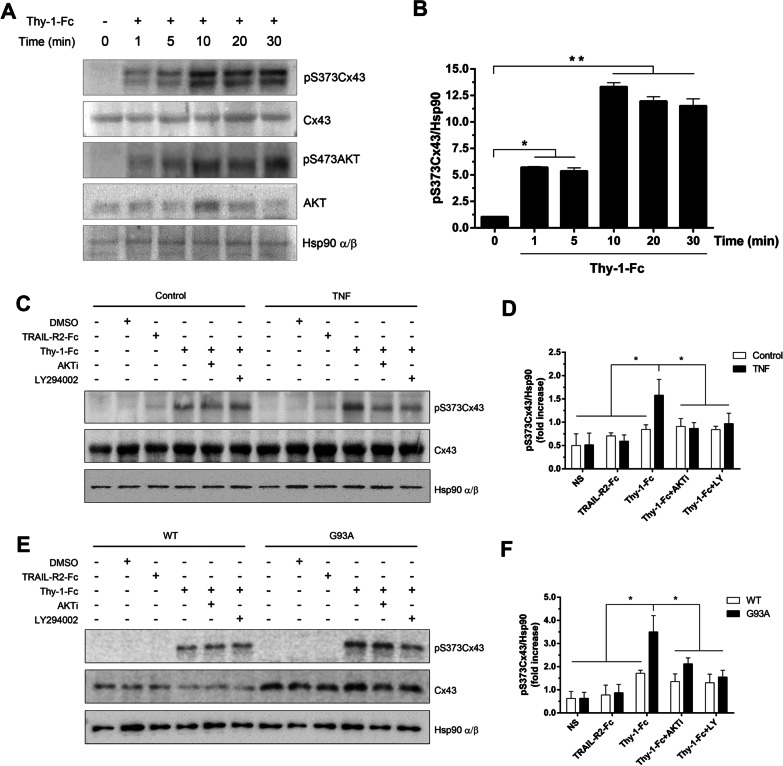
Levels of phospho-Cx43 in Thy-1-stimulated astrocytes under proinflammatory conditions. **A** Kinetics of changes in pS373Cx43, Cx43, pS473AKT and AKT protein levels in Thy-1-Fc-stimulated primary rat astrocytes previously treated for 48 h with TNF (10 ng/ml). Hsp90 α/β was used as a loading control. **B** Quantification of the average values for the pS373Cx43/Cx43 ratio obtained by densitometric data analysis of the results shown in (**A**). Values are mean ± s.e.m. from 3 separate experiments and indicate the ratio between the densitometric values of the bands and the respective Hsp90 α/β control value. Astrocytes derived from rat brains, treated or not with TNF for 48 h (**C**, **D**). Astrocytes derived from hSOD1^G93A^ or hSOD1^WT^ mice (**E**, **F**) incubated for 30 min with either the vehicle (0.03% DMSO), the AKT inhibitor (AKTi, 3 μM), or the PI3K inhibitor LY294002 (LY, 3 μM). pS373Cx43 protein levels were evaluated by immunoblotting analysis of extracts from cells stimulated with Thy-1-Fc for 10 min or treated with TRAIL-R2-Fc as a control. **D**, **F** Quantification of the average pS373Cx43/Hsp90 α/β ratio values obtained by densitometric data analysis of the results shown in **C** and **E**, respectively. Values are mean ± s.e.m. from 3 independent experiments *p<0.05, **p<0.01 (ANOVA, Bonferroni post-test)

Thy-1 activates the PI3K/AKT pathway in astrocytes by engaging the α_v_β_3_ integrin [36]. Since S373Cx43 is a known downstream target of AKT [51] and Cx43 levels increase in reactive astrocytes (Fig. 2B), we evaluated S373Cx43 phosphorylation (pS373Cx43) after stimulating the TNF-treated astrocytes with Thy-1. We found that pS373Cx43 levels increased significantly after 10 min and remained elevated up to 30 min (Fig. 3A, B). As a control for activation of Thy-1-α_v_β_3_ integrin signaling, we evaluated S473AKT phosphorylation levels (pS473AKT) for the same period of time, as shown for pS373Cx43. As expected [36], pS473AKT levels increased at all time points tested (Fig. 3A). We additionally assessed Cx43 phosphorylation after Thy-1 stimulation for 10 min in astrocytes derived from rat brains treated or not with TNF (10 ng/ml, 48 h, Fig. 3C, D). Additionally, astrocytes derived from hSOD1^G93A^ transgenic mice, a pathological model of ALS, were compared with their hSOD1^WT^ counterparts (WT) (Fig. 3E, F). Furthermore, we employed PI3K and AKT inhibitors (LY294002 and AKTi, respectively) to evaluate the participation of the PI3K/AKT signaling pathway in Cx43 phosphorylation. We observed an increase in pS373Cx43 after stimulating with Thy-1 (Fig. 3C–F), but not in the respective controls (control in Fig. 3C and WT in Fig. 3E). This effect was precluded with the AKT and PI3K inhibitors (Fig. 3C–F) only in TNF-treated (Fig. 3C, D) and hSOD1^G93A^-derived astrocytes (Fig. 3E, F), suggesting a role for the PI3K/AKT signaling axis in Cx43 phosphorylation induced by inflammation and Thy-1. Treatment with the vehicle (DMSO in Fig. 3C and E) or TRAIL-R2-Fc did not affect pS373Cx43 levels (Fig. 3C–F). TRAIL-R2-Fc was used as a negative control and accounts for possible non-specific effects caused by the Fc portion of the fusion proteins [2, 36, 37]. As expected, cells treated with TRAIL-R2-Fc behave as in non-stimulated cells (NS in Fig. 3D and F).

**Fig. 4:**
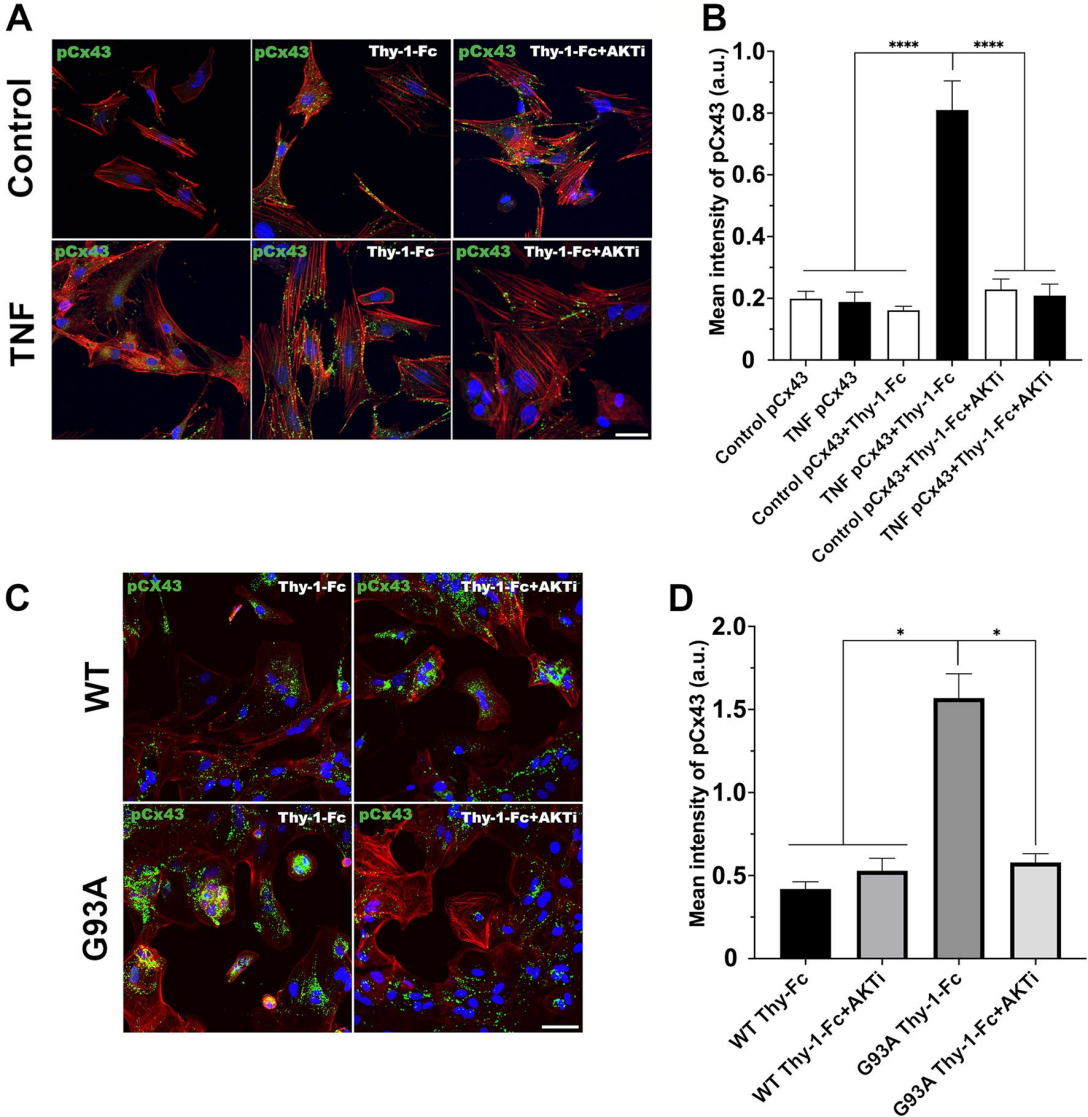
PhosphoS373Cx43 levels in Thy-1-stimulated reactive astrocytes. Representative immunofluorescence microphotographs of rat astrocytes treated or not with TNF (**A**), and astrocytes from transgenic hSOD1^WT^ (WT) or hSOD1^G93A^ (G93A) mice (**C**), stimulated or not with Thy-1-Fc for 10 min to evaluate Cx43 phosphorylation (green). Cells were also incubated or not with the AKT inhibitor (AKTi, 3 μM). Magnification bar = 50 μm. **B**, **D** Values in the graphs are the quantification of mean intensity of phosphoS373Cx43 (pCx43) fluorescence normalized by mean intensity of DAPI (blue) fluorescence expressed in arbitrary units (a.u.). Twenty-five cells were evaluated per condition, Control versus TNF (**B**) and WT versus G93A (**D**) with or without AKTi treatment before Thy-1 stimulation. *p < 0.05 (Mann–Whitney test)

We used the same experimental conditions to evaluate the effect of Thy-1 on Cx43 phosphorylation by indirect immunofluorescence analysis. Here, non-treated/non-stimulated astrocytes and non-treated astrocytes stimulated with Thy-1 (control with no TNF + Thy-1) were used as negative controls. We have previously reported that non-reactive astrocytes do not respond to Thy-1 stimulation [37, 38]. Therefore, reactive and non-reactive rat astrocytes and astrocytes derived from the ALS mouse model, or the respective WT control model were stimulated with Thy-1-Fc (Thy-1) with or without pretreatment with the AKT inhibitor (AKTi). Upon stimulation with Thy-1, increased pS373Cx43 staining was observed in reactive astrocytes (+ TNF) obtained from the rat brain, compared with the control cells (non-TNF treated; Fig. 4A, left panels). A similar result was found in the mouse ALS model (G93A), compared to the respective control (WT; Fig. 4C, left panels). Under the same Thy-1-stimulated conditions but using cells pretreated with AKTi, pS373Cx43 levels were reduced in both TNF/Thy-1-treated rat brain astrocytes (Fig. 4A, right panels) and in G93A astrocytes (Fig. 4C, right panels). These observations were confirmed by quantifying the mean fluorescence intensity of pS373Cx43, normalized by nuclear staining, using the Fiji ImageJ software (Fig. 4B, D). Therefore, these results correlate with those observed by immunoblotting analysis (Fig. 3) and indicate that under inflammatory conditions and Thy-1 stimulation, Cx43 is a downstream target of the PI3K/AKT signaling axis.

### AKT-mediated phosphorylation of Cx43 regulates ATP release

**Fig. 5:**
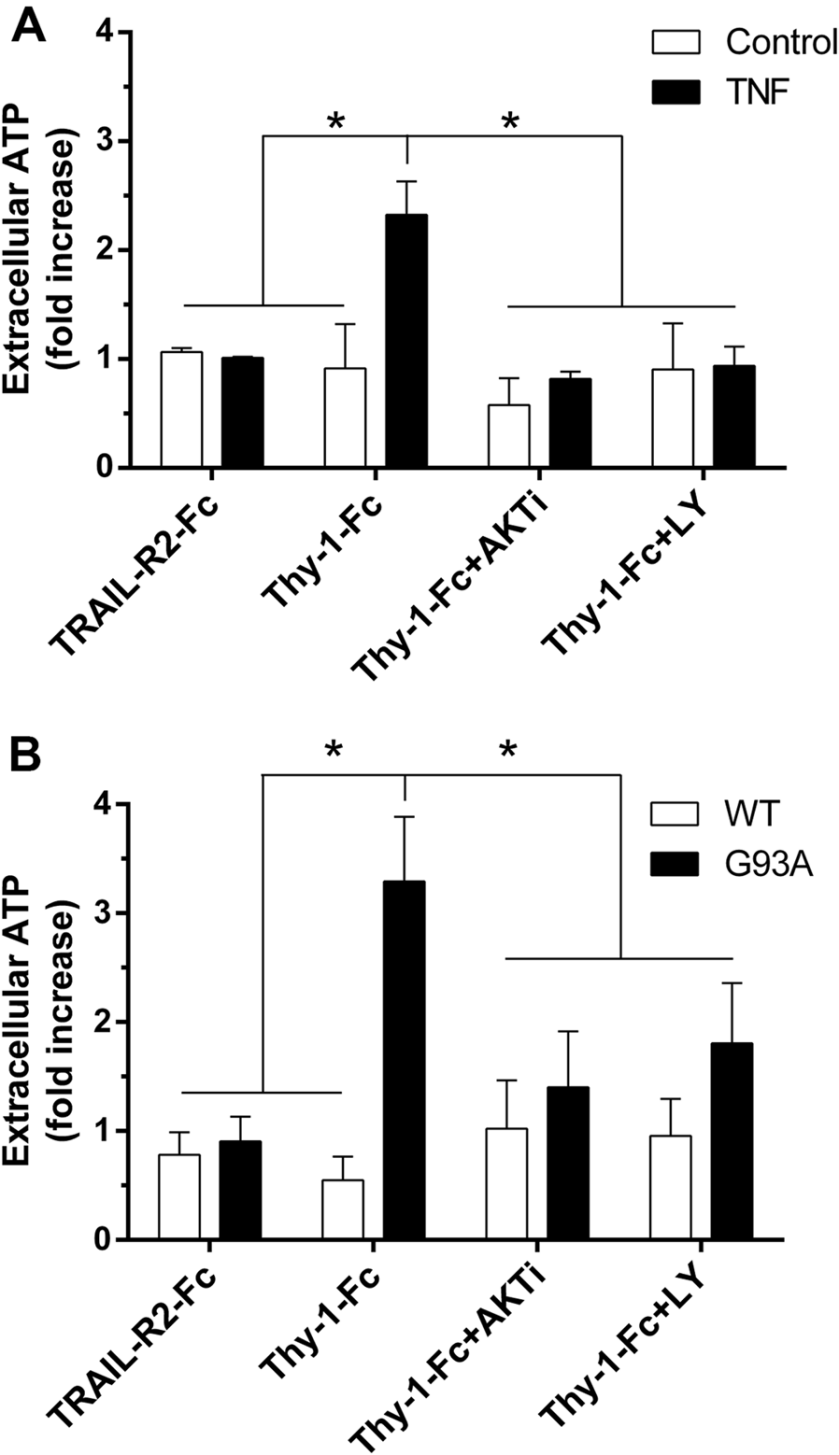
Thy1-induced release of ATP depends on PI3K/AKT activation in reactive astrocytes. ATP measurements in the extracellular medium of non-reactive (Control) or reactive (TNF, 10 ng/ml, 48 h) primary rat astrocytes (**A**), as well as astrocytes derived from hSOD1^G93A^ (G93A) or hSOD1^WT^ control mice (**B**). Cells were pre-treated with vehicle (0.03% DMSO), the AKT inhibitor (AKTi, 3 μM) or the PI3K inhibitor LY294002 (LY, 3 μM) for 30 min, and then stimulated with Thy-1-Fc or TRAIL-R2-Fc for 10 min. Values are mean ± s.e.m. from 3 independent experiments, *p<0.05 (ANOVA, Bonferroni post-test)

Reactive astrocytes stimulated with Thy-1 release ATP by opening Cx43 HC [2, 37]. Therefore, as a functional assay for HC opening, we evaluated ATP release by rat- and ALS mouse-derived astrocytes. Our previous results indicated that maximum release of ATP due to Thy-1 stimulation occurs after 10 min [37]. Therefore, we tested the effect of the aforementioned PI3K/AKT inhibitors under the reported conditions. As expected, Thy-1 stimulation significantly increased ATP release in reactive astrocytes (+ TNF), compared to the control condition without TNF treatment. This effect was not observed when the PI3K/AKT pathway inhibitors were used (Fig. 5A). Likewise, astrocytes derived from the ALS mouse model (hSOD1^G93A^) released significantly higher ATP levels in response to Thy-1, compared to the corresponding control (hSOD1^WT^). This effect was prevented by treatment with either AKTi or LY294002 (Fig. 5B), as seen for rat astrocytes (Fig. 5A). Altogether, these results confirm the direct participation of the PI3K/AKT pathway in HC opening and ATP release from astrocytes stimulated with Thy-1.

### In vivo phosphorylation of Cx43 by AKT under inflammatory conditions

**Fig. 6:**
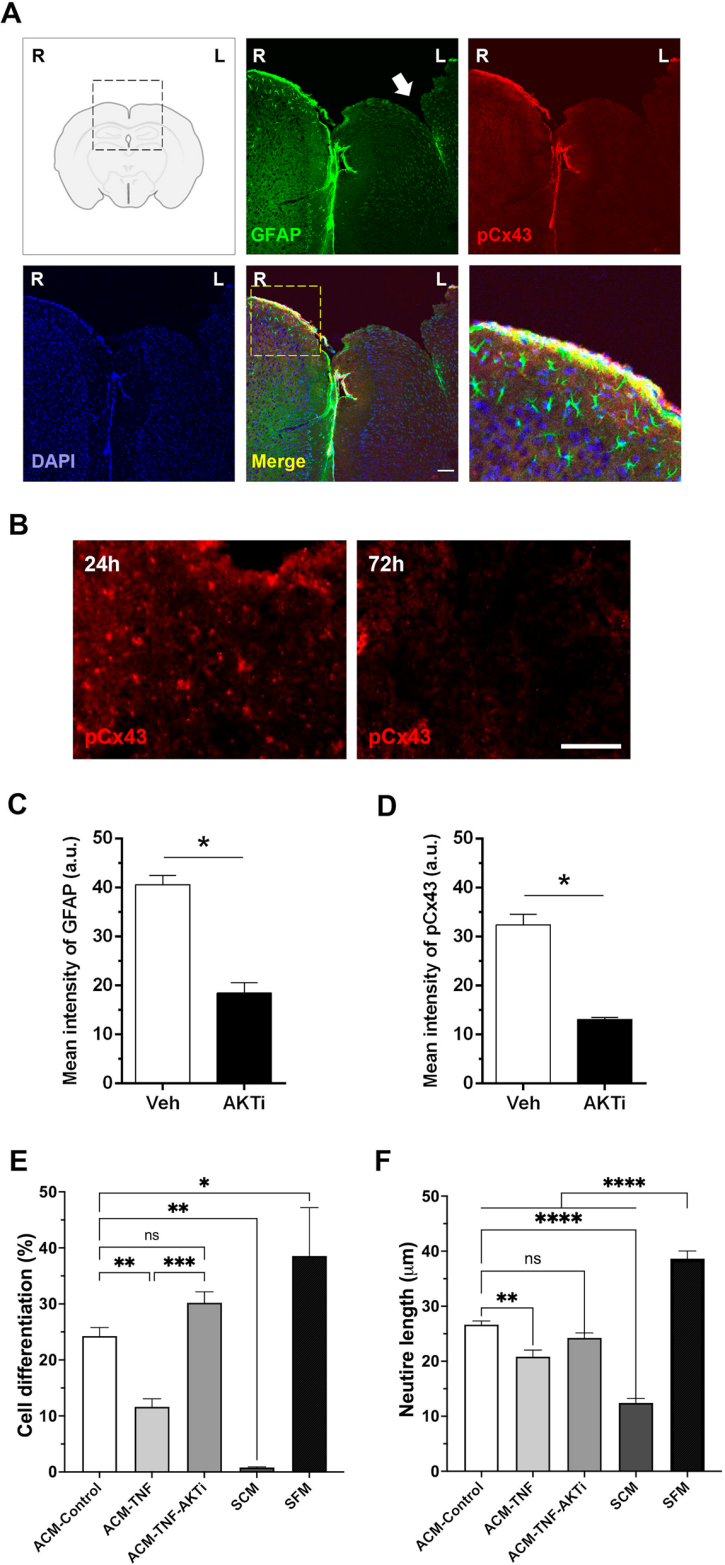
In vivo assay of Cx43 phosphorylation in mouse cortex and in vitro treatment of astrocytes with AKTi to protect neurons from damage. Coronal section of the right (R, Vehicle) and left-brain hemisphere (L, AKTi) of C57BL/6 J male mice injured by the insertion of a needle in both hemispheres. Mice were injected with the vehicle in R (DMSO) or the AKT inhibitor (AKTi, 60 nmol) in L, and killed 24 h or 72 h post-surgery. **A** Brain tissue sections were labeled with anti-GFAP (green) antibodies, anti-pS373Cx43 (red) antibodies, or DAPI (nuclei, blue) at 72 h. Magnification bar = 50 μm. A threefold digital zoom was applied to the yellow dash square-marked area in the merged picture and appears to the right of the panels. **B** Brain tissue sections labeled with anti-pS373Cx43 (red) antibodies at 24 h and 72 h. Values in the graphs are the quantification of the mean intensity of GFAP (**C**) or of pCx43 in arbitrary units (a.u.) (**D**). **E**, **F** Quantification of two different morphological parameters (% differentiation and neurite length) using the NeuroJ software (ImageJ, USA) on bright-field microscopy images. CAD cells were differentiated for 48 h in serum-free medium (SFM) containing sodium selenite or undifferentiated by keeping them in serum-containing medium (SCM). Other conditions included were differentiated CAD cells treated with medium previously conditioned by astrocytes left only in SFM (ACM control), or treated with TNF or with TNF + AKTi for 48 h. These astrocytes were incubated 5 days in SFM to obtain the ACM. Percentage of differentiated CAD cells with processes > 15 µm (**E**); average length of the processes extended by differentiated cells in microns (**F**). Values are mean ± s.e.m. from 3 separate experiments, *p < 0.05 (Mann–Whitney test)

In this in vivo assay, we locally damaged the brain of WT mice by bilaterally inserting a needle into the cortex. The left hemisphere was injected with AKTi, whereas the right hemisphere received only the vehicle. Twenty-four and 72 h post-surgery, we obtained brain tissue sections and employed immunofluorescence assays to evaluate S373Cx43 phosphorylation (24 h) and GFAP staining (72 h) under inflammatory conditions, with or without AKTi treatment (Fig. 6). GFAP staining was assessed in both hemispheres as a marker of astrogliosis (Fig. 6A). The pS373Cx43 label was higher after 24 h than after 72 h in the right hemisphere (Fig. 6B). GFAP staining of higher intensity was more evident in the right hemisphere (R), compared to the left one (L) (Fig. 6A, C). Furthermore, the pS373Cx43 label was clearly decreased in the left hemisphere (L) where AKTi had been added, compared to the contralateral right hemisphere (R), where greater phosphorylation of Cx43 was detected (Fig. 6A, D). Noteworthy, the increased pS373Cx43 staining overlapped with that of GFAP, suggesting that astrocytes are likely to be the cells with elevated levels of active Cx43. These results indicate that under inflammatory conditions in vivo, AKT phosphorylates Cx43 in astrocytes, thereby confirming our in vitro observations.

Notably, these in vivo experiments indicate that AKTi treatment decreased GFAP staining, and therefore, astrocyte reactivity, in the treated (L) hemisphere. We reasoned that, if AKTi reduced the inflammatory effect of wounding that leads to astrocyte reactivity, neurons would also be protected by the presence of this inhibitor. To test this idea in vitro, we obtained conditioned media from astrocytes (ACM) treated or not with TNF, in the presence or absence of AKTi. The effect of these ACM was assessed by adding them to differentiated CAD cells. We have previously reported that, apart from killing neurons (Fig. 2C, D), reactive astrocytes also inhibit neurite outgrowth and induce retraction of neuronal processes of neurons differentiated in culture [29]. Here, we found that neurons treated with ACM from control astrocytes exhibited on average 24.3 ± 1.5% of differentiated neurons, with an average length of 26.7 ± 0.7 µm (white bars, Fig. 6E, F), while those treated with ACM from reactive astrocytes (ACM-TNF) showed lower percentage of differentiated neurons and shorter neurites (11.6 ± 1.4% and 20.8 ± 1.2 µm, respectively; Fig. 6E, F). As expected, the effect of ACM from astrocytes treated with TNF and AKTi resembled that of ACM from control astrocytes, showing on average 30.2 ± 2% differentiation and processes of 24.3 ± 0.9 µm in length (Fig. 6E, F). In this experiment, the undifferentiated and differentiated controls were neurons treated with serum-containing medium (0.8 ± 0.1% differentiation, 12.4 ± 0.8 µm) and SFM containing sodium selenite (38.6 ± 8.6% differentiation, 38.7 ± 1.4 µm), respectively (black bars, Fig. 6E, F). The results indicate that AKTi decreases the proinflammatory effect of TNF on astrocytes, thereby allowing them to maintain their supportive effect towards neurons.

## Discussion

A correlation between upregulation of the PI3K/AKT pathway and astrocyte reactivity has been previously reported [16], and a role for Cx43 activation in astrocyte reactivity is deemed important in astrogliosis [67, 72]; however, a connection between these events has not been previously established. In addition, the possible upstream ligand–receptor interactions that trigger these signaling events have not been defined yet. In the present study, we provide evidence that the Thy-1-α_v_β_3_ integrin interaction in reactive astrocytes activates the upregulated AKT, and that this kinase phosphorylates the S373 residue of Cx43, which reportedly leads to HC opening [9, 62] and the release of ATP. Therefore, the modulation of AKT and Cx43 HC activation represent a means to control the adverse effects of astrogliosis.

In a cellular model of primary astrocytes under inflammatory conditions, we provide evidence here for the upregulation of PI3K/AKT pathway components (Fig. 2A, B). These results coincide with the in silico data obtained by comparing several reactive astrocyte databases (Fig. 1). Moreover, reactive astrocytes from the cerebral cortex (TNF-treated or hSOD1^G93A^) respond to Thy-1—a neuronal glycoprotein that binds to α_v_β_3_ integrin and Syndecan-4—by activating AKT, which phosphorylates Cx43 (Figs. 3 and 4) and leads to the release of ATP to the extracellular medium (Fig. 5). Since Cx43 acts as a direct AKT target in Thy-1-stimulated reactive astrocytes, we propose that Cx43 plays an important role in neuron–astrocyte communication through the regulation of extracellular ATP levels.

In reactive astrocytes, the levels of various proteins increase, including GFAP, vimentin, nestin and inducible nitric oxide synthase (iNOS) [17, 37, 47], as well as cell adhesion molecules, such as Syndecan-4 and α_v_β_3_ integrin [26, 33, 37]. Under inflammatory conditions, TNF reprograms the astrocytes to augment expression of plasma membrane receptor proteins and ion channels related to adhesion and subsequent migration, including Cx43, Pannexin1 and the P2X7R [37]. When Thy-1 binds to α_v_β_3_ integrin, the engaged receptors trigger an intracellular signaling cascade that increases intracellular Ca^2+^ concentration and activates the non-receptor tyrosine kinases FAK and Src. These kinases then recruit several other proteins, including PI3K, paxillin, vinculin, and p130Cas, which are part of the multimolecular adhesome complex [6, 36, 41, 69]. Furthermore, elevated cytosolic Ca^2+^ leads to the opening of Cx43 HCs, ATP release and P2X7R activation, which further increases intracellular Ca^2+^ concentration, leading to cell migration [2, 27, 37]. Importantly, Thy-1-induced cellular responses triggered via the α_v_β_3_ integrin/FAK/PI3K/Cx43/ATP/P2X7R signaling pathway only occur in TNF-treated or reactive astrocytes.

The bioinformatics analysis of this study identified a large number of altered genes in the different sets of selected databases (Table 1 and Additional file 2: Table S2). Among the pathways with the most altered genes were those involving PI3K/AKT, Regulation of Actin Cytoskeleton, and FA. The present study, as well as our previous reports [2, 4, 36], validate these results. The PI3K/AKT pathway has great relevance in cellular processes such as survival, proliferation, migration and neuroprotection [31, 46]. We previously reported that the PI3K/AKT pathway participates in the adhesion and migration of reactive astrocytes [2, 36]. Alternatively, other studies implicated this pathway in the regulation of the inflammatory state in response to different types of CNS damage [16, 42, 75]. For example, in primary rat cortical astrocytes subjected to a mechanical strain, increased levels of S473AKT phosphorylation and rapid ATP release are observed [49]. This stretch-induced AKT phosphorylation is attenuated by blocking Ca^2+^ influx and PI3K, suggesting that the PI3K/AKT signaling pathway may play a relevant role in the maintenance of reactive gliosis after trauma [49]. Additionally, Salas et al. reported that metabolic inhibition induces Cx43 HC permeability in an AKT-dependent manner [62]. According to our current in vitro and in vivo findings, phosphorylation of Cx43 by AKT in an injury model could account for ATP release. Therefore, we posit that pS373Cx43 represents the missing link between PI3K/AKT activation and elevated ATP levels in the extracellular medium.

Another important aspect regarding the phenotype of reactive astrocytes after spinal cord injury is astrocyte proliferation, which is promoted by the brain-derived neurotrophic factor (BDNF) and regulated by PI3K/AKT signaling [74]. Therefore, controlling the expression and activity of this axis could serve to prevent the proliferation of reactive astrocytes after spinal cord injury and preclude formation of the glial scar, which should favor axonal regeneration. Since the PI3K/AKT pathway also participates in astrocyte migration [36], the use of inhibitors of this pathway could serve as another approach to mitigate the formation of the glial scar. For example, Phosphatase and Tensin homologue deleted on Chromosome 10 (PTEN) is an important, physiologically relevant negative regulator. In a spinal cord injury model, PTEN overexpression in astrocytes attenuates glial scar formation and gliosis, leading to improved locomotor function and axon regeneration at the lesion site [15].

In the present study, stimulation of primary reactive astrocytes from the cerebral cortex with Thy-1 led to activation of the upregulated PI3K/AKT/Cx43 pathway and phosphorylation of the Cx43 S373 residue. Cx43 activation results in opening of the HCs [62] and subsequent ATP release to the extracellular medium [37]. Additionally, inhibition of the PI3K/AKT pathway prevented the release of ATP (Fig. 5). Therefore, considering that Cx43 HC opening is required for ATP release [2, 37], and that both Cx43 HC opening and ATP release are controlled by PI3K/AKT activation, we suggest that the Thy-1/α_v_β_3_ integrin/PI3K/AKT/Cx43/ATP release signaling axis represents an important sequence of events participating in astrogliosis.

On the other hand, elevated extracellular ATP exacerbates neuronal responses by obliging them to adjust to greater Ca^2+^ influxes due to the over-stimulation of purinergic receptors, which ultimately can cause cell death [19]. In a pathophysiological context, we also evaluated the activation of the PI3K/AKT pathway using astrocytes from the hSOD1^G93A^ transgenic ALS mouse model, where the increased levels of Cx43 in astrocytes represent an inherent trait [1], and the correlation between astrocyte reactivity and neurodegenerative processes is well documented [7]. We previously reported that these astrocytes express high levels of Cx43, β_3_ integrin and P2X7R [37]. Here, we demonstrated that after Thy-1 stimulation, astrocytes increase Cx43 phosphorylation by AKT (Figs. 3 and 4) and significantly increase extracellular ATP levels (Fig. 5). These results could partially explain the toxic levels of ATP observed in the ALS brain microenvironment, which likely activate purinergic receptors. Consequently, activation of P2 receptors, together with other cellular factors, might finally lead to neuronal death, as observed in the brains of ALS mice [3, 59].

In our in vivo model, where mice were injected with AKTi, we found that Cx43 phosphorylation levels in the cerebral cortex decreased 24 h post-surgery. In this model, general neocortex inflammation was elevated, and pS373Cx43 and GFAP levels were significantly higher than in the control hemisphere injected only with the vehicle (Fig. 6). Hence, using different in vitro and in vivo reactive astrocyte models, we demonstrate here that the PI3K/AKT/Cx43 pathway is important in neuroinflammatory processes. Noteworthy, the in vivo results provide evidence that in the presence of the AKTi, brain injury leads to a 50% reduction in GFAP levels, compared to the control hemisphere without the AKTi (Fig. 6C), suggesting decreased astrogliosis and consequently less neuronal damage. This fact is supported by in vitro experiments that reveal the harmful effect of the conditioned medium obtained from reactive astrocytes (TNF-treated) on neurons, an effect that is not observed when astrocytes are treated with TNF in the presence of the AKTi (Fig. 6D, E). These results indicate that AKT inhibition reduces the neuronal toxicity caused by reactive astrocytes, and therefore, aiming to this network represents a promising therapeutic target to control astrocyte reactivity and the progression of neurodegenerative diseases.

## Conclusions

In astrogliosis, the expression of genes in the PI3K/AKT molecular interacting network is altered. Additionally, the Thy-1-α_v_β_3_ integrin interaction in reactive astrocytes increases AKT activity and the phosphorylation of S373Cx43, thereby promoting HC opening and the release of ATP. Therefore, therapeutic strategies to prevent AKT and Cx43 HC activation represent attractive approaches to control the adverse effects of astrogliosis.

## Supplementary Information

**Additional file 1: Table S1.** Datasets used in the in silico analysis. Lists of the Dataset Series (GSE) and Platform (GPL) accession numbers used in this study. The number of probes and samples per dataset are indicated. The “Treatments” section briefly describes the microarrays used to identify the DEG in the various conditions leading to reactive gliosis, compared to their respective controls.

**Additional file 2: Table S2.** Up- and downregulated genes grouped in three signal transduction cascades. The number of up- and downregulated genes in the different sets of selected databases are presented for those pathways showing high numbers of altered genes: the PI3K/AKT, Regulation of Actin Cytoskeleton, and Focal Adhesion signaling cascades.

## Data Availability

The datasets analyzed in the current study are available in the Gene Expression Omnibus (GEO) repository of PubMed, https://www.ncbi.nlm.nih.gov/geo/. All data generated or analyzed as part of this study are included in this article and its supplementary information files.

## Abbreviations

ACM: Astrocyte-conditioned medium
ATP: Adenosine trisphosphate
ALS: Amyotrophic lateral sclerosis
CAD: Cath.a-differentiated
CNS: Central nervous system
Cx43: Connexin43
ECM: Extracellular matrix
HCs: Hemichannels
FA: Focal adhesions
GFAP: Glial fibrillary acidic protein
PI3K/AKT: Phosphatidylinositol 3-kinase/protein kinase B
P2X7R: P2X7 receptor
SFM: Serum-free medium
SOD1: Superoxide dismutase 1
TNF: Tumor necrosis factor
