# Supplementary figures and images for: Protein kinase B (AKT) upregulation and Thy-1-αvβ3 integrin-induced phosphorylation of Connexin43 by activated AKT in astrogliosis
Source: J Neuroinflammation. 2023 Jan 6;20:5. doi: 10.1186/s12974-022-02677-7 (PMC9817390; doi:10.1186/s12974-022-02677-7)

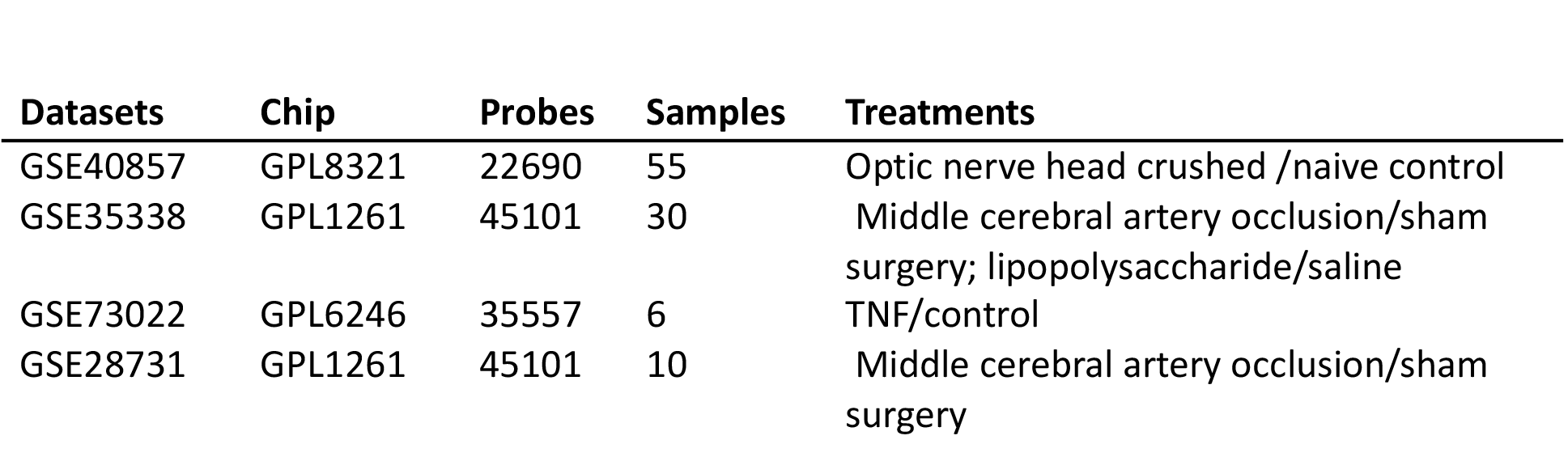

Supplement: Supplementary file 1 — Additional file 1: Table S1. Datasets used in the in silico analysis. Lists of the Dataset Series (GSE) and Platform (GPL) accession numbers used in this study. The number of probes and samples per dataset are indicated. The “Treatments” section briefly describes the microarrays used to identify the DEG in the various conditions leading to reactive gliosis, compared to their respective controls. [file 12974_2022_2677_MOESM1_ESM.tif]

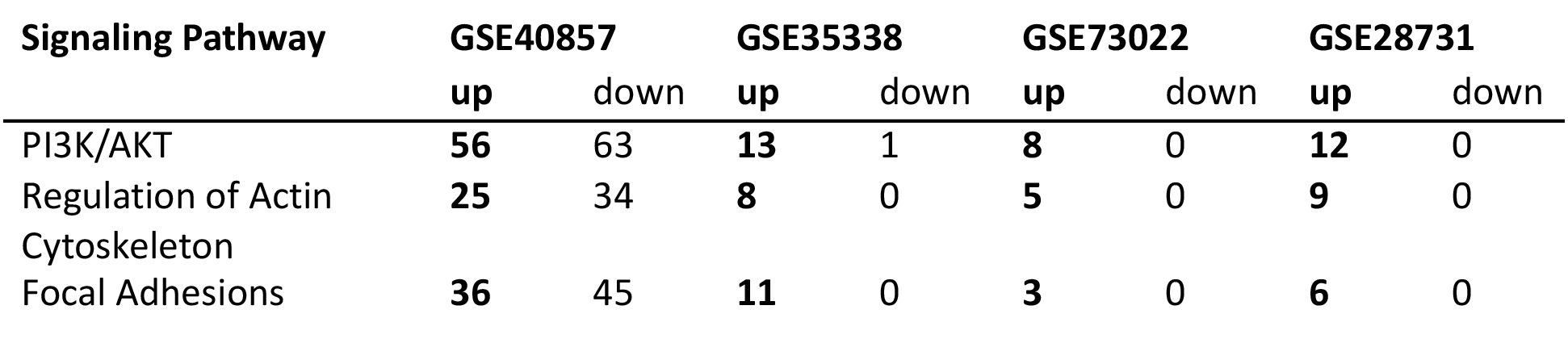

Supplement: Supplementary file 2 — Additional file 2: Table S2. Up- and downregulated genes grouped in three signal transduction cascades. The number of up- and downregulated genes in the different sets of selected databases are presented for those pathways showing high numbers of altered genes: the PI3K/AKT, Regulation of Actin Cytoskeleton, and Focal Adhesion signaling cascades. [file 12974_2022_2677_MOESM2_ESM.tif]
